# A Mimicker of Malignancy: A Case Report on Elastofibroma Dorsi

**DOI:** 10.7759/cureus.83079

**Published:** 2025-04-27

**Authors:** Annie Pham, Kristina McLeod-van Amstel, Gillian Redlich, Elie Barakat

**Affiliations:** 1 Radiology, Edward Via College of Osteopathic Medicine, Alexandria, USA; 2 Interventional Radiology, Edward Via College of Osteopathic Medicine, Alexandria, USA; 3 Pathology, CHRISTUS St. Frances Cabrini Hospital, Alexandria, USA; 4 Interventional Radiology, Edward Via College of Osteopathic Medicine, Monroe, USA

**Keywords:** ct scan, elastofibroma dorsi, infrascapular, pet scan, pseudotumor

## Abstract

Elastofibroma dorsi is a soft tissue tumor rarely identified on imaging. It is most commonly located in the infrascapular region, deep to the serratus anterior and latissimus dorsi of older women. We present a case of an asymptomatic female patient with a history of left rotator cuff repair and chronic lymphocytic leukemia in remission who was found to have elastofibroma dorsi. As a benign tumoral entity that can look hypermetabolic on positron emission tomography-computed tomography scan (PET-CT), this case demonstrates the importance of an effective clinical diagnosis of elastofibroma dorsi to avoid unnecessary procedures and their related complications while also providing an enhanced understanding of its clinical presentation and radiological and pathological features.

## Introduction

Elastofibroma dorsi was first described by Jarvi and Saxen in 1959 at the Congress of Scandinavian Pathologists [1]. It is a rare benign soft tissue tumor typically located in the infrascapular region, deep to the serratus anterior and latissimus dorsi, and is thought to occur from recurrent friction in the area between the inferior scapula and the posterior chest wall [2,3]. Demographically, elastofibroma dorsi is seen in up to 2% of the population, particularly in older women, with a mean age of diagnosis between 65 and 70 years [2,3].

A higher index of suspicion for this tumoral entity can be made following clinical and imaging findings [3]. Clinically, on exam, elastofibroma dorsi has no firm attachments to overlying skin but is challenging to distinguish from the surrounding anatomical structures [4]. In more than 50% of cases, elastofibroma dorsi are characterized by an asymptomatic evolution with a tendency to grow slowly over time [3]; however, up to 50% of patients may alternatively report pain or a clicking sensation in the scapula [2].

On computed tomography scan (CT), it presents as a poorly defined mass with attenuation similar to that of adjacent muscle. It has been suggested that smaller elastofibromas appear more homogeneous on CT as compared to larger ones [2]. This homogenous appearance has also been reported when using thoracic ultrasound [4].

Pathologically, it is characterized by fibrous tissue, spindle cells, collagenized stroma, and an excess amount of scattered abnormal elastic fibers, which appear as densely eosinophilic, irregular, variably sized structures that may include entrapped tissue [2].

These tumors may show mild to moderate uptake on fluorodeoxyglucose (FDG) positron emission tomography-computed tomography scan (PET-CT), which can be mistaken for malignancy. Of note, mean standardized uptake values (SUVs) lie between 1.4 and 3.2 for elastofibroma dorsi [5]. Interestingly, SUVs for malignancy are generally greater than 2.5, which contributes to possible misdiagnosis [6].

Treatment options range from observation to surgical resection [1]. It should be recognized that surgery is typically only pursued for symptomatic cases and/or cases where the fibroelastoma dorsi is significant in size [5,7]. Additionally, surgical removal does pose potential postoperative complications such as hematoma, wound seroma, and prolonged chronic pain [2,4,8]. In the case of incomplete removal, local recurrence of the tumor has also been shown [2,4]. Morbidity rates following surgery have been shown to vary from 11% to 44%, depending on which case series are accounted for in the statistical analysis [8].

## Case presentation

**Figure 1:**
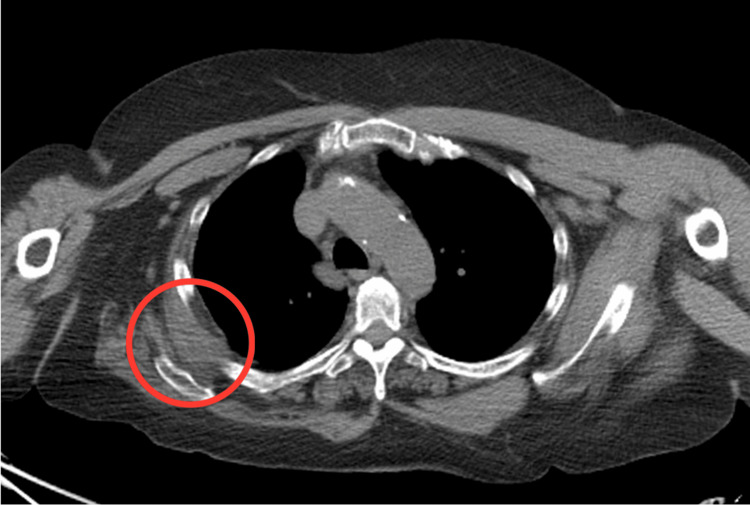
Chest CT (plain) revealing a soft tissue mass (red circle) along the lower angle of the scapula and subjacent ribs

We present a case of a 69-year-old Caucasian female with a history of left rotator cuff repair, chronic lymphocytic leukemia in remission, and squamous cell carcinoma status post right upper lobe resection. On review of systems and physical exam, the patient reported she was doing well with no complaints and had a normal physical exam. On routine follow-up chest CT, a soft tissue mass was noted in the right upper posterior chest wall between the lower angle of the scapula and the subjacent ribs (Figure 1).

**Figure 2:**
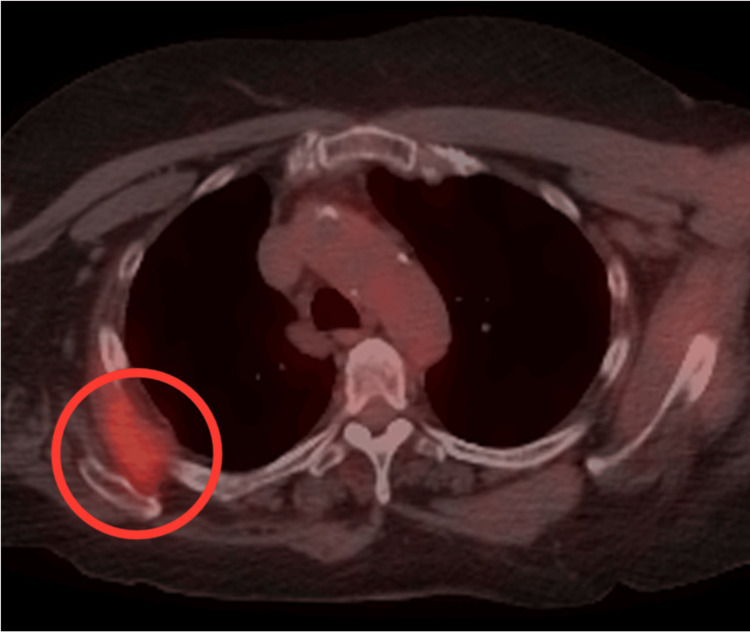
Chest PET-CT showing soft tissue uptake in the periphery of the right upper posterior chest wall (red circle), between the lower angle of the scapula and the subjacent ribs, with a maximum SUV of 3.9. PET-CT: positron emission tomography-computed tomography scan; SUV: standardized uptake value

Given the patient’s history of cancer, a chest PET-CT was obtained, revealing an area of hypermetabolism in the periphery of the right upper posterior chest wall, between the lower angle of the scapula and the subjacent ribs (Figure 2).

**Figure 3:**
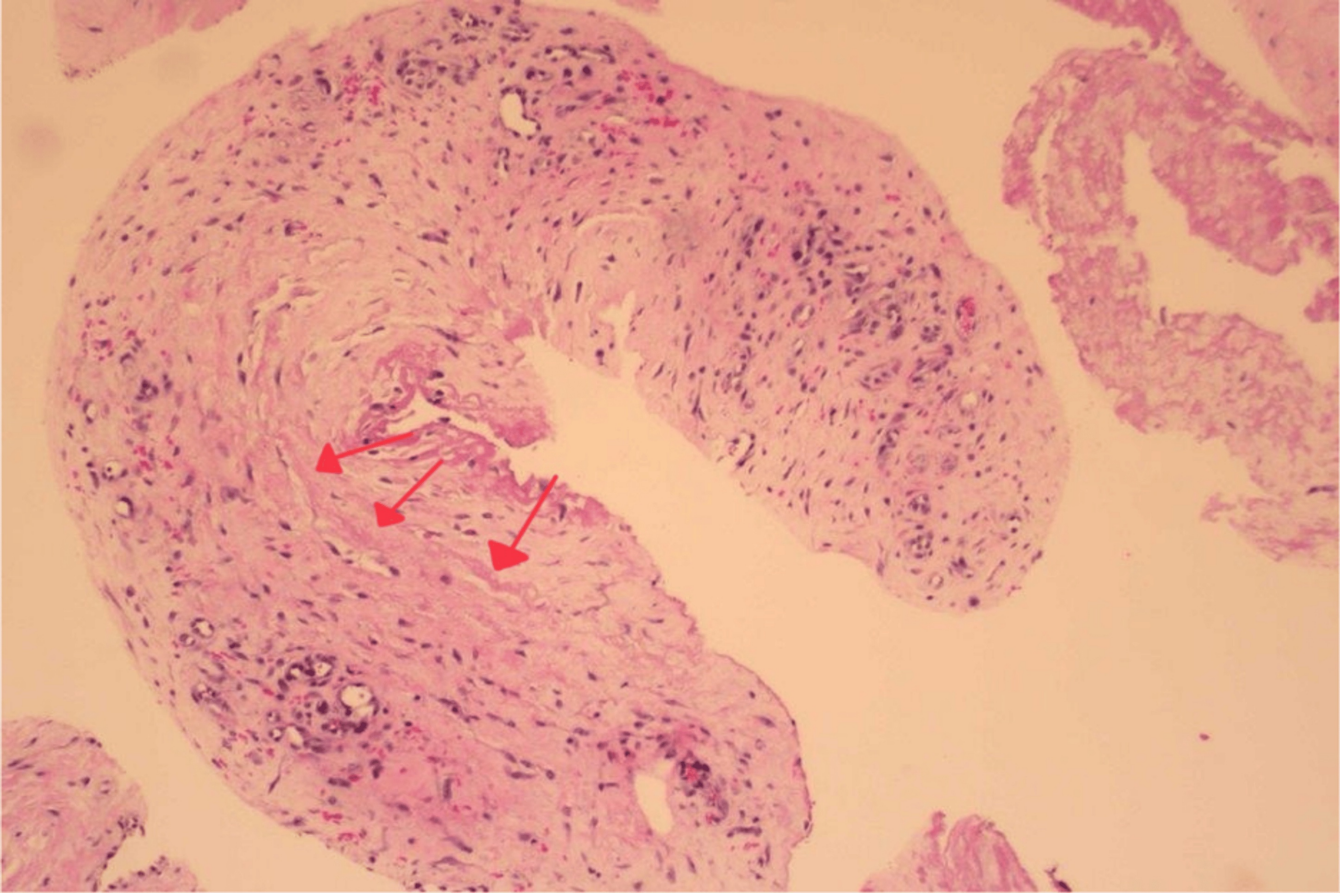
Histology shows scattered abnormal elastic fibers appearing as densely eosinophilic, variably sized structures (red arrows) at 40X magnification

**Figure 4:**
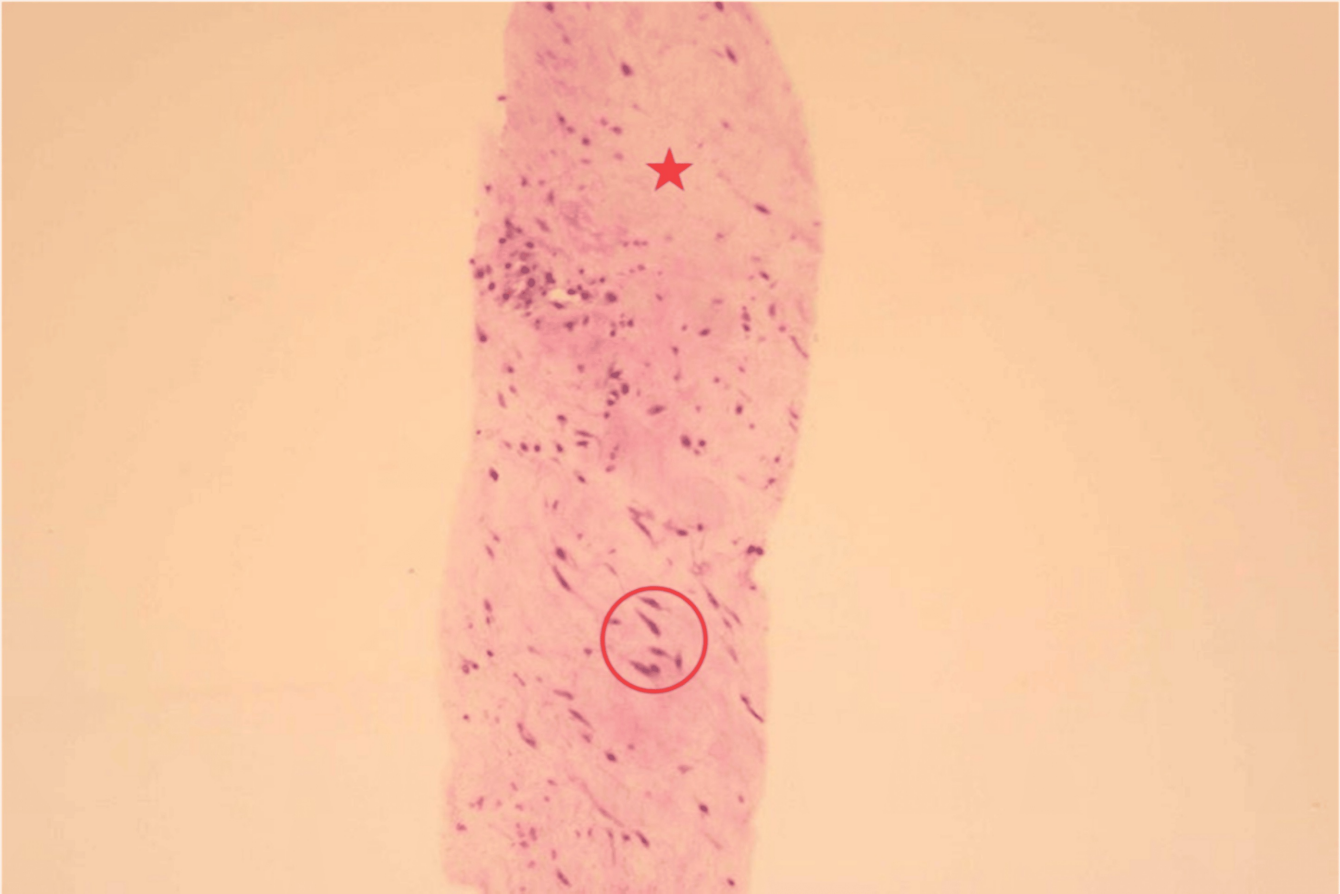
Histology shows benign spindle cells (red circle) and collagenized stroma (red star) at 10X magnification

This area of FDG uptake had not been present in prior imaging and was investigated further via a biopsy. Pathologically, the specimen obtained showed scattered abnormal elastic fibers as densely variable-sized structures at 40X magnification, as well as benign spindle cells and collagenized stroma at 10X magnification (Figures 3, 4).

Based on the findings, the patient was subsequently diagnosed with fibroelastoma dorsi. Treatment options at this point were discussed with the patient, who opted for no intervention but continued routine cancer screenings as needed per her oncology team.

## Discussion

While this patient did fit within the typical demographics of elastofibroma dorsi, most commonly affecting elderly women in their sixth to eighth decades of life, it should be noted that demographics alone are not sufficient to rule out this tumoral entity. Elastofibroma dorsi is seen in up to 2% of the population, particularly in older women, with a mean age of diagnosis between 65 and 70 years. Cases have also been reported in individuals as young as six years old, as well as in some patients in their fourth decade of life [4,9]. Elastofibroma dorsi typically arises in the infrascapular region, situated deep to the serratus anterior and latissimus dorsi muscles. While unilateral lesions are more common (most frequently on the right side), approximately 30% of cases present bilaterally. These lesions are characterized by an asymptomatic evolution and tend to grow slowly in more than 50% of cases. Although often asymptomatic, up to 50% of patients may report pain or a clicking sensation in the scapula [2].

Additionally, PET-CT does not necessarily need to be pursued if prior imaging and clinical findings are consistent with elastofibroma dorsi [4]. These tumors may exhibit mild to moderate uptake on FDG PET-CT, which can be mistaken for malignancy. Reported cases have shown SUVs ranging between 1.52 and 1.98 [2]. In our case, the patient did not report any signs or symptoms of cancer; however, their recent history of malignancy was taken into consideration during medical decision-making, and PET-CT followed by biopsy was performed. Notably, this case demonstrated a higher maximum SUV of 3.9 on PET-CT compared to the averages reported in prior studies [5]. Other imaging modalities that may be considered include MRI, which is regarded as the most sensitive and specific imaging tool for diagnosing elastofibroma dorsi. However, MRI is not ideal for evaluating malignancy [4,10,11]. Histological findings from the biopsy in this case, including eosinophilic abnormal elastic fibers, benign spindle cells, adipose tissue, and collagenous tissue, were consistent with those reported in the literature, thereby confirming the diagnosis [2,12,13].

Regarding treatment, the patient opted against surgical intervention. Currently, elastofibroma dorsi is considered to have no malignant potential, as there have been no cases of malignant proliferation following elastofibroma dorsi described in the literature. However, there have been several reported cases of local recurrence following incomplete surgical removal of the entire tumoral tissue [3,14]. Furthermore, the patient was asymptomatic and the lesion measured less than 5 cm in diameter, which falls below the typical threshold for surgical removal [4,5].

## Conclusions

This case highlights the importance of radiologists and oncologists recognizing the characteristic imaging features and clinical presentation of elastofibroma dorsi. Familiarity with this benign soft tissue lesion can help prevent misdiagnosis and unnecessary interventions. Ultimately, accurately identifying elastofibroma dorsi ensures appropriate management and reduces patient anxiety.
